# Angiopoetin-like 4 in sickle cell retinopathy

**DOI:** 10.1042/BSR20181462

**Published:** 2018-10-31

**Authors:** Ricardo Luz Leitão Guerra, Mariana Gouveia Bastos, Cristina Salles

**Affiliations:** 1 Bahiana’s School of Medicine and Public Health, Salvador, Brazil; 2 Retina Division, Clínica de Olhos Leitão Guerra, Salvador, Brazil

**Keywords:** angiopoietin 4, angiogenic proteins, sickle cell

## Abstract

This correspondence provides a comment on the recent review article by Yang et al. [*Biosci. Rep.* (2018) **38**, BSR20180557, https://doi.org/10.1042/BSR20180557]

We read the article by Yang et al. [1] with great interest. The study assembled valuable information regarding regulatory effects of angiopoietin-like 4 (ANGPTL4)-associated pathways and provided information for the potential development of this protein as a clinical treatment target in eye disease therapy [1].

We have some considerations about their study.

The aqueous humor (AH) is an intraocular fluid, secreted by the ciliary body that fills both the posterior and anterior chambers of the eye [2,3]. The vitreous body [4], also known as vitreous humor (VH), is a transparent gel that comprehends 80% of the eye volume [3].

The terminology *in aqueous* means ‘containing water as a solvent or medium’ and *in vitro* means ‘elsewhere outside a living organism’.

In their article, Yang et al. [1] used the terminologies ‘in aqueous’ and ‘*in vitro*’ referring to Jee et al. [5] research regarding sickle cell retinopathy (SCR). Reading Yang et al. study, we understood that Jee et al. [5] performed a research outside a living organism [1].

However, Jee et al. [5] studied samples of the AH and VH and noticed that the expression of ANGPTL4 was increased in patients with SCR, suggesting that ANGPTL4 might be related to the development of retinal neovascularization in SCR and could therefore be a therapeutic target for the treatment of proliferative SCR.

We believe that the considerations aforementioned might help in understanding the valuable scientific information assembled by Yang et al. [1].

We celebrate Yang et al. [1] for the presentation and offer our respects.

## Abbreviations

AH: aqueous humour
ANGPTL4: angiopoietin-like 4
SCR: sickle cell retinopathy
VH: vitreous humour
